# Methods for Identification of CA125 from Ovarian Cancer Ascites by High Resolution Mass Spectrometry

**DOI:** 10.3390/ijms13089942

**Published:** 2012-08-09

**Authors:** Florian Weiland, Katarina Fritz, Martin K. Oehler, Peter Hoffmann

**Affiliations:** 1 Adelaide Proteomics Centre, School of Molecular and Biomedical Science, University of Adelaide; Adelaide 5005, Australia; E-Mails: florian.weiland@adelaide.edu.au (F.W.); katarina.fritz@medunigraz.at (K.F.); 2 Research Centre for Reproductive Health, Robinson Institute, University of Adelaide; Adelaide 5005, Australia; E-Mail: martin.oehler@adelaide.edu.au

**Keywords:** CA125, mucin 16, mass spectrometry, ovarian cancer, antibody, false-positives

## Abstract

CA125 is the most widely used tumour marker in ovarian cancer with unsatisfactory sensitivity and specificity especially at early stage. It is quantified by antibody-based immunoassays; however different molecular weight isoforms have been described in the literature which have never been validated by mass spectrometry, potentially affecting the diagnostic accuracy and clinical reliability of the test. In this study, CA125 was detected by Western blot and its identity confirmed by mass spectrometry. Two-dimensional (2D) gel electrophoresis in combination with mass spectrometry revealed that positive Western blot signals up to 500 kDa are most likely false-positive interactions of M11-like and OC125-like antibodies. Fibronectin, identified as one of these false-positive interaction partners, increased the reading for CA125 in a first generation ELISA significantly (*p* = 0.02). The existence of low-molecular weight isoforms of CA125 is therefore questionable and is most likely reflecting cross-reactivity of the antibodies with other proteins. This would explain the conflicting reports on the molecular structure of CA125 and also the inconsistency of CA125 levels by different ELISAs. Our results are also the first steps towards a mass spectrometric assay for CA125 quantification, which would improve sensitivity and reliability.

## 1. Introduction

Cancer antigen 125 (CA125), also known as mucin-16 (MUC16), is the most widely used tumour marker in ovarian cancer, and considered the “gold standard”. Serum level of CA125 is used to monitor response to chemotherapy, relapse, and disease progression in ovarian cancer patients. However, its role for screening and early detection of ovarian cancer is limited due to a low sensitivity and specificity. Nevertheless, it is used for diagnostic purposes in combination with other methods such as transvaginal ultrasonography [1].

CA125 was first detected by *Bast et al.* in 1981 using a murine monoclonal antibody purified following immunization of mice with a human ovarian cancer cell line [2]. This antibody reacted with the majority of ovarian carcinoma cells and appeared to be nonreactive with non-malignant tissues. The antibody was called OC125 and the corresponding class of antibodies that recognize the same epitope are known as OC125-like antibodies. To date, several different classes of antibodies recognizing CA125 are known, with OC125-like and M11-like antibodies the most frequently used. These antibodies provide the basis for CA125-detection by enzyme linked immunosorbent assay (ELISA). First generation ELISAs use the same antibody for catching and tracing of the antigen, which renders a repetition of the epitope necessary. In contrast, second generation assays use OC125-like antibodies as catchers and M11-like antibodies as tracers and *vice versa. Davelaar et al.* [3] tested both first and second generation assays, concluding that both provide results with concordant tendencies; however, for monitoring a single patient, assays should not be interchanged due to varying values within the different assays. The cut-off value established for CA125 levels is 35 kU/L [4]. However, various malignant and benign conditions such as pregnancy, cardiovascular and liver diseases give rise to elevated CA125 levels [5–7]. Due to this lack of specificity, CA125 levels are only considered for monitoring treatment response and follow-up of ovarian cancer patients.

Thus far, characterization of CA125 has relied almost exclusively on the usage of antibodies. CA125 forms with molecular masses ranging from 110 kDa to more than 2000 kDa have been reported [8–13]. The protein has commonly been identified in the interface of stacking and separating gel, which points to a very high molecular weight [14,15]. Identification of CA125 by mass spectrometry has been described very rarely in literature [16–18]. High accuracy MS and MS/MS data is only available for CA125 with a mass of 2–3 MDa [19], for lower molecular weight forms no such data is available.

Here we present high accuracy MS and MS/MS data for CA125 and our findings question the existence of molecular mass isoforms below 500 kDa. Furthermore, the reliability of CA125 detection by probing with antibodies is challenged as we identified several antigens interacting with the M11-like and OC125-like antibody resulting in false-positive signals. One of those antigens, fibronectin, elevated the CA125 reading significantly in a first generation M11-like ELISA. The reliability of first generation ELISA to determine CA125 levels is therefore doubtful. All together, the findings explain the conflicting reports on the molecular structure of CA125 (reviewed in [20]) and also the inconsistencies of CA125 levels measured by different ELISAs [3].

## 2. Results

### 2.1. One-Dimensional (1D) Gel Electrophoresis of Human Ascites

As the various molecular mass isoforms of CA125 span over a range of 110–2000 kDa [8–13], we used a *T* = 3–8% polyacrylamide gel to resolve all forms simultaneously. A total of 94 μg protein from patient (P) 517 ascites was loaded onto a 1D gel. The Western blots showed positive signals from the interaction of the M11-like antibody at molecular masses of approximately 117 kDa, 200 kDa, 250 kDa, 460 kDa, 500 kDa and a positive signal at the edge of the sample well (Figure S1). These protein bands were excised from a Coomassie stained gel (Figure S1) and subjected to identification by mass spectrometry (Table S1). With this approach we were not able to identify CA125 by mass-spectrometry in any excised bands.

### 2.2. Enrichment of CA125 by Size Exclusion Chromatography

**Figure 1:**
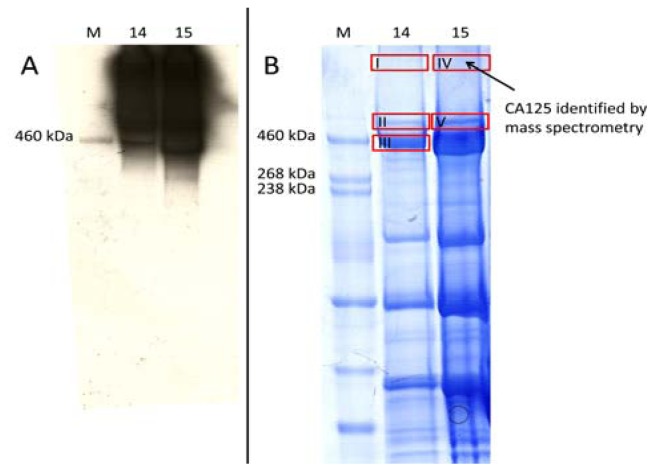
(**A**) Western blot of SDS-PAGE (*T* = 3–8%) of size exclusion chromatography (SEC) fractions 14 and 15, M indicates the molecular mass marker lane; (**B**) Coomassie brilliant blue stained SDS PAGE (*T* = 3–8%) gel of SEC fractions 14 and 15. M indicates the molecular mass marker lane, roman numbers indicate bands corresponding to positive signals in western blot, which were subsequently identified by mass-spectrometry, CA125 was identified in Band IV by mass spectrometry (see Tables 1 and S1).

In order to identify CA125 by mass spectrometry, we applied size exclusion chromatography (SEC) (7) to reduce sample complexity and to enrich CA125. Ascites of P86 was chosen for SEC as it has a higher level of CA125 in comparison to P517. 500 μL of ascites was separated by SEC and the derived fractions were tested by dot-blotting for reactivity with the M11-like antibody (Figure S2). Fraction 14 and 15 showed the highest intensity signal for CA125. Then 200 μL of each fraction was precipitated with a ReadyPrep 2D clean-up kit. Proteins from each fraction were separated on a 1D gel and electro-blotted (Figure 1A). The bands showing positive signals were excised from a Coomassie stained 1D gel (Figure 1B) and identified by mass spectrometry (Table S2). Analysis by tandem mass spectrometry of band IV on the very top of the 1D gel identified twenty-one unique peptide sequences of CA 125; five of them exhibited MASCOT scores over the significance threshold (Expectation ≤ 0.05, see Tables 1 and S2). However, CA125 could not be identified in the excised bands I, II, III and V.

### 2.3. Two-Dimensional (2D) Electrophoresis of Human Ascites

**Figure 2:**
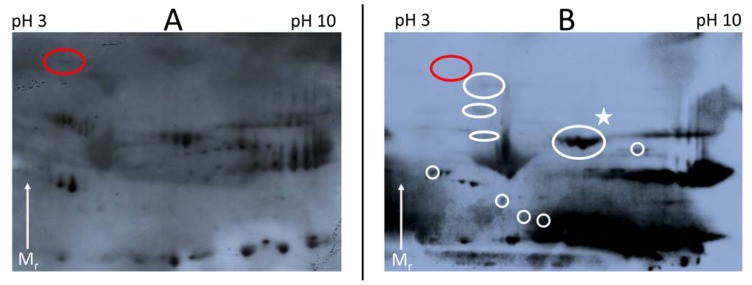

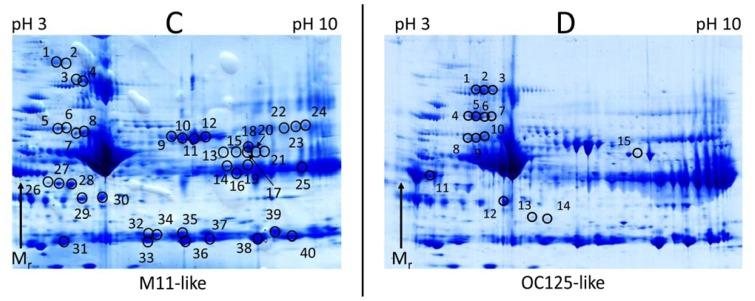
(**A** and **B**): 2D Western blot (225 μg protein load, P517 ascites, IPG 3-10NL, *T* = 4–12%), probed with HRP conjugated M11-like antibody (**A**) or OC125-like antibody and secondary HRP-conjugated antibody (**B**). White circles in (**B**) correspond to signals exclusive in their interaction with OC125-like antibody. (**C** and **D**): Coomassie stained 2D gel (225 μg protein load, P517 ascites, IPG 3-10NL, *T* = 4–12%), numbers indicate protein spots subjected to identification by mass spectrometry (see Tables S3 and S4 respectively); these spots co-locate with the signals from the western blots shown in Figure 2A (**C**) or Figure 2B (**D**) respectively. The star in Figure 2B indicates proteins already indentified in Figure 2C, corresponding to protein spots 9–12. The red circle in Figure 2A indicates the signal obtained by a M11-like antibody (subsequently identified as fibronectin (see Table S3); the red circle in Figure 2B indicates the missing signal for fibronectin when probing with an OC125-like antibody.

Two-Dimensional gel electrophoresis (2DE) facilitates the separation of proteins by their isoelectric points (pI) and their molecular weights, thus we hypothesized that this might separate CA125 isoforms from other proteins with equivalent molecular weight. As 2DE offers a higher protein loading capacity than 1D SDS-PAGE, we used 225 μg of protein isolated from P517 ascites without prior CA125 enrichment by SEC. The sample was labeled with Cy2 and separated by pI in the range of pH 3–10. Second dimension was performed in a *T* = 4–12% SDS-PAGE gel. The proteins from the 2D gel were electroblotted onto a low-fluorescent PVDF membrane, scanned with an Ettan DIGE Imager and afterwards proteins interacting with the M11-like and OC125-like antibodies were detected by ECL (Figure 2(A,B)). By overlaying the two images we were able to locate the proteins which gave a positive signal accurately. Corresponding proteins were excised from a Coomassie stained 2D gel (225 μg protein load, Figure 2(C,D)) and identified by mass spectrometry (Tables S3 and S4). The results of the identification showed that CA125 could not be identified from these 2D gels.

### 2.4. Elisa of Human Serum Spiked with Fibronectin

**Figure 3:**
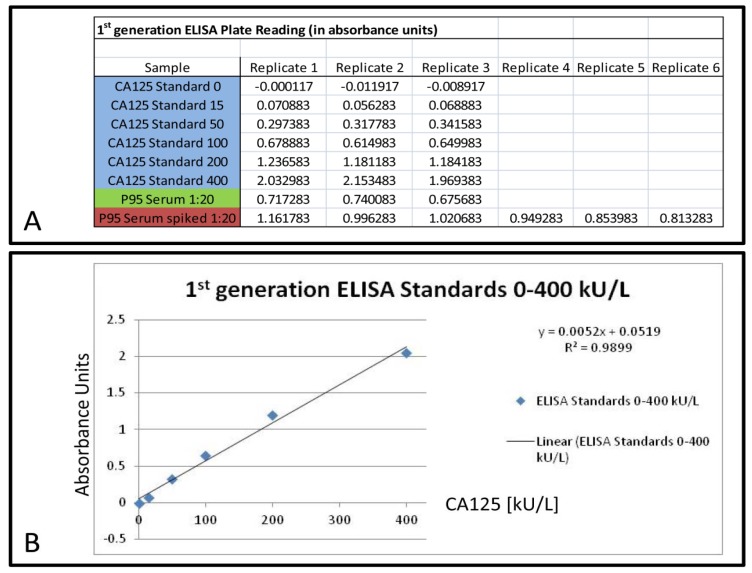

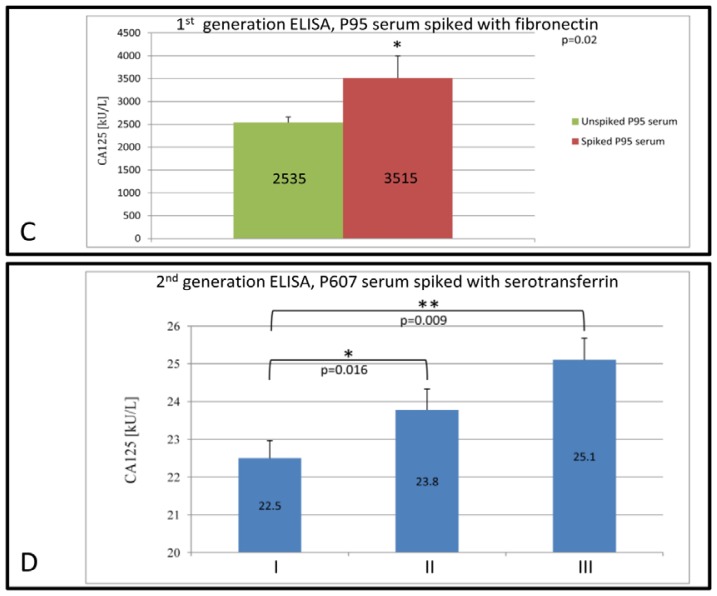
(**A**) Measured values in absorbance units for CA125 standards (0–400 kU/L) (blue), 1:20 diluted P95 serum (green) and 1:20 diluted P95 serum spiked with 80 μg fibronectin (red). The values obtained from P95 serum and spiked P95 serum were used for the Mann-Whitney U test; (**B**) Standard curve obtained by plotting the detected absorbance units against the kU/L of the CA125 standards. This curve was used to calculate the CA125 concentration of the used samples **(C)** CA125 concentration measured by a first generation ELISA (M11-like). Green: P95 serum, red: P95 serum spiked with 80 μg fibronectin. The increase in reading is statistically significant (* *p* < 0.05). Error bars indicate standard deviation of measured values within the respective sample; (**D**) CA125 concentrations measured by a clinically used second generation ELISA. I: 500 μL P607 serum + 100 μL H_2_O; II: 500 μL P607 serum + 100 μL apo-serotransferrin (100 mg/mL); III: 500 μL P607 serum + 100 μL holo-serotransferrin (100 mg/mL). The increase in the reading for CA125 is statistically significant (*) between apo-serotransferrin (II) and unspiked serum (I) and statistically highly significant (** *p* < 0.01) between holo-serotransferrin (III) and unspiked serum (I). Error bars indicate the standard deviation of the measured values in the respective sample.

To verify that these false-positive signals on the 2D western blot were not derived from CA125 co-localization, but were resulting from an interaction between the M11-like antibody and those protein identified by mass spectrometry, fibronectin was chosen for spiking experiments. This protein was selected as it was identified in 1D gel bands and 2D gel protein spots by MS (1D: Figure S1, Table S1 (Band III and IV), 2D: Figure 2(A,C) (Spot number 1 and 2, Table S3) and was commercially available. We spiked 80 μg of fibronectin into 100 μL of 1:20 diluted P95 serum (as this M11-like-only ELISA Kit, according to the manufacturer, is only suitable for serum) and performed an ELISA for CA125. Accordingly, the spiked fibronectin represented ~2.3% of the total protein amount, based on a serum concentration of 7000 mg/dL. Normal serum levels of fibronectin are approximately 200 μg/mL [21], meaning the spiked fibronectin levels were in a 80-fold excess (on a 1:20 dilution of serum basis, this equals approximately a 4-fold excess in undiluted serum). We chose this unrealistically high excess as we were assuming that normal fibronectin amounts do not increase the CA125 reading significantly, given that serum from healthy people has a CA125 level below 35 kU/L. A Mann-Whitney U-test showed that the spiked serum has a statistical significantly (*p* = 0.02) (see Figure 3C) increased reading for CA125 in a M11-like-only sandwich ELISA by an average of 980 kU/L (compared to the unspiked P95 serum). This means that a 1-fold excess of fibronectin raises the CA125 reading by around 12 kU/L, thus normal serum levels of fibronectin are below the critical reading of 35 kU/L. To test the influence of fibronectin on a clinically used M11-like and OC125-like sandwich ELISA, we repeated above mentioned experiment with a clinically approved sandwich ELISA, but no increased CA125 reading was detected (data not shown).

### 2.5. Elisa of Human Serum Spiked with Serotransferrin

Serotransferrin was detected as a protein giving a false-positive signal with both M11-like and OC125-like antibodies (see Figure 2). Therefore it was hypothesized that it would increase the CA125 reading in a clinically used, second generation ELISA. Based on a normal serotransferrin serum concentration of 2.04–3.60 mg/mL [22], 500 μL of P607 serum was spiked with 10 mg apo-serotransferrin and holo-serotransferrin respectively, which equals an approximate 5-fold increase of this protein. The samples were given to the Institute of Medical and Veterinary Sciences, Adelaide (IMVS) to measure the CA125 concentration. Both samples with spiked serotransferin showed a statistically significant increase in CA125 reading (apo-serotransferrin: *p* = 0.016; holo-serotransferrin: *p* = 0.009); however, the increase was only minor. The unspiked P607 serum had a CA125 reading of 22.5 kU/L, the P607 serum spiked with apo- and holo-serotransferrin showed a CA125 reading of 23.8 and 25.1 kU/L, respectively (see Figure 3D).

### 2.6. High Accuracy Mass Spectrometry of CA125

Mass spectrometric data for CA125 has rarely been reported and MS/MS data is nearly non-existent in the literature. To obtain high accuracy mass spectrometric data we performed a filter aided digest of the SEC fractions 13–17, as they gave positive signals when probed with an M11-like antibody (see Figure S2). Before analyzing these samples on a LTQ Orbitrap XL mass spectrometer, all digested fractions were tested for LC-MS incompatible polymer contamination with MALDI mass spectrometry and subsequently fraction 14 had to be excluded from high accuracy MS (data not shown). We chose the Orbitrap for this task as this mass spectrometer can achieve a mass accuracy <5 ppm with a 95% probability [23]. CA125 could be identified in fractions 15–17 (see Table 2). The combined data of those fractions displays 32 unique peptides of CA125; while 16 of them have a MASCOT score which is above the significance threshold (Expectation ≤ 0.05), the mass accuracy is in the range of 0.03 to 2.25 ppm (see Table 2, MS/MS spectra of statistically significant peptides in Figure S3). The other 16 are despite their lower score likely CA125 peptides because they were measured with high accuracy between 0.15 and 3.53 ppm, which is a similar accuracy to the 16 peptides above the threshold.

## 3. Discussion

The current standard procedure for the detection of CA125 in biofluids is SEC in combination with 1D-SDS-PAGE, western-blotting and probing with M11-like and/or OC125 type of antibodies [18,24]. Reports on the verification of CA125 signals by mass spectrometry are rare and lack detail [16–19]. This is especially unsatisfactory for CA125, as it is a very large protein and this raises the probability for random peptide matches, particularly as no statistical scoring is given. Here we report the mass spectrometric identification of CA125 as a high molecular weight protein from a 1D gel after enrichment by denaturing SEC (see Figure 1 and Table 1). Additionally, the positive SEC fractions have been digested by trypsin and analyzed by an LTQ Orbitrap XL to obtain high accuracy mass spectrometric and MS/MS data of CA125. We identified 16 unique peptides of CA125; another 16 peptides were identified, but have MASCOT scores below the significance threshold (see Table 2). However, as we enriched for CA125 it is likely that these peptides are real discoveries and not random matches because of the high mass accuracy of the measurement (between 0.15 and 3.53 ppm). The mass accuracy of the identified significant peptides was equal or better than 2.25 ppm (see Table 2); the false discovery rate on the peptide level in the fractions 15, 16 and 17 was 1.65%, 1.85% and 2.07% respectively. These findings make it evident that it is possible to acquire sufficient MS data to verify signals from probing with anti-CA125 antibodies, instead of relying on immuno-blots alone. This is especially important as we show that there are cross-reactions with the M11-like antibody in at least one case. The positive signals observed on the 2D Western-blots (see Figure 2, Table S3 and S4) are caused by mostly the same proteins we also identified in a 1D gel (see Figure S1 and Table S1). Potentially, the low molecular weight forms of CA125 could be masked by co-migrating proteins in 1D and 2D gels or otherwise impossible to detect by mass spectrometry. However, we show that spiking of human serum with fibronectin increases the reading for CA125 significantly (*p* = 0.02) in a first generation M11-like ELISA (see Figure 3C). This confirms the existing of at least one false-positive interaction partner of M11-like antibodies. Spiking 80 μg of fibronectin into 1:20 diluted serum resulted in an increase in the CA125 concentration measured by a first generation ELISA by 980 kU/L. Therefore, the amount of spiked fibronectin raises the reading for CA125 in undiluted serum by only one twentieth of the measured increase, *i.e.*, 49 kU/L (increase in reading divided by dilution factor). This would raise CA125 readings, in a first generation ELISA, over the clinically used cut-off value of 35 kU/L. Importantly, as 80 μg of fibronectin equals to an 4-fold excess in undiluted serum, a normal (1-fold) serum level of fibronectin, would contribute around 12 kU/L to the CA125 level, according to our experiment. This is below the established cut-off of 35 kU/L.

These findings raise reasonable doubt about the molecular properties of published low-molecular weight isoforms of CA125. Based on our results, it cannot be excluded that proteins cross-reacting with the CA125 antibodies have been characterized along the primary protein of interest. This may have lead to the mutually exclusive characteristics of CA125 in terms of carbohydrate content and predominant type of glycosylation [8,20,25]. Therefore, mass spectrometry is an indispensible tool for assessing the purity of CA125 preparations. The observed secondary reactions of the applied antibodies also raise questions about the low molecular weight isoforms of CA125. These forms have been identified so far exclusively by antibody probing and therefore independent evidence of CA125 identity is required.

Furthermore, we show that OC125-like and M11-like antibodies cross-react with different protein subsets (see Figure 2(A,B)). This provides one explanation for the varying levels of CA125 detected by different ELISAs [3] as distinct antibodies against CA125 are utilized. The reaction of the M11-like and OC125-like antibodies to different protein subsets gives also an explanation to the failure of fibronectin to raise the CA125 readings in a second generation ELISA, which employs M11-like and OC125-like antibodies. Fibronectin is only an interaction partner with M11-like antibodies, as spots 1 and 2 in Figure 2C (identified as fibronectin, see Tables S3 and S4) have no corresponding signal in Figure 2B. Serotransferrin was identified as a secondary interaction partner for M11-like and OC125-like antibodies and was able to raise the reading for CA125 in a second generation ELISA statistically significantly, however only minor slightly (Figure 3D). The reason for this is unknown, however it can be speculated that the cross-reaction of the different antibody types in the respective anti-CA125 antibody families is varying and the here used M11-like and OC125-like antibodies do not match the antibody strains applied by the IMVS. Therefore, this result should have only little impact on the clinical detection measurement of CA125. However, it can be argued that an increase in serum concentration of several proteins, affecting readings in the CA125 assay, could be cumulative and have a more significant impact on the clinical detection.

## 4. Experimental Section

This study was approved by the Human Ethics Committee of Adelaide University.

### 4.1. Sample Preparation

Ascites of two ovarian cancer patients (P517 and P86) was collected and submitted to the IMVS to determine the amount of CA125. The remaining ascites was aliquoted and stored at −80 °C. The CA125 concentrations of P517 and P86 ascites were 6609 kU/L and 36,000 kU/L, respectively.

Blood of ovarian cancer patients P95 and P607 was collected into clotting tubes (Greiner Bio-one), centrifuged at 3000 rpm for 10 min at room-temperature and the plasma was removed and stored in 500 μL aliquots at −80 °C.

### 4.2. 2D Electrophoresis

Proteins from P517 ascites were precipitated using the ReadyPrep 2D clean up kit (Bio-Rad Laboratories) according to manufacturer’s protocol and resuspended in TUC4% buffer (7 M urea, 2 M thiourea, 4% (3-[(3-Cholamidopropyl)-dimethylammonio]-1-propansulfonat (CHAPS), 2% Dithiothreitol (DTT), 2% Pharmalyte 3-10 (GE Healthcare, Buckinghamshire, UK, 30 mM Tris). The protein concentration was estimated using EZQ protein quantitation kit (Life technologies Corporation, Carlsbad, CA, USA). Prior to isoelectric focusing, 225 μg of protein was labeled with Cy2 (GE Healthcare) according to manufacturer’s protocol, deviating in the use of 200 pmol of CyDye label for 225 μg of proteins, the labeling was omitted when the proteins were stained with Coomassie brilliant blue after 2D electrophoresis. IPG strips were rehydrated overnight in TUX 1% buffer (6 M Urea, 2 M Thiourea, 50% Acetonitrile, 1% CHAPS, 0.5 Pharmalyte 3–10 (GE Healthcare) and 200 mM 2,2-Dithiodiethanol). Isoelectric focusing was performed on an IPGphor II (GE Healthcare) using anodic cup loading according to published protocols [26]. SDS-PAGE was carried out on Criterion XT gels (Bio-Rad Laboratories, Hercules, CA, USA) with a *T*-value (*T*) of 4–12% according to manufacturer’s protocol. Proteins were stained with Coomassie brilliant blue overnight.

### 4.3. Western Blot

After SDS-PAGE, proteins were electroblotted onto a low-fluorescent PVDF membrane (Merck KGaA, Darmstadt, Germany) using a Criterion wet blotter (Bio-Rad). The membrane was then scanned on an Ettan DIGE imager (GE Healthcare) using the Cy2 channel. Two different anti-CA125 antibodies were used. In the first work-flow, the membrane was incubated with directly horse-radish peroxidase (HRP)-conjugated monoclonal anti-CA125 antibody (M11-like) (HyTest, Joukahaisenkatu, Finland). In the second work-flow the membrane was incubated with a mouse derived monoclonal OC125-like antibody (Thermo Fisher Scientific, Waltham, MA, USA), afterwards the membrane was incubated with an anti-mouse HRP-conjugated IgG (Rockland Immunochemicals, Gilbertsville, PA, USA). Both work-flows applied enhanced chemiluminescence (ECL) detection (Sigma-Aldrich, St. Louis, MO, USA).

### 4.4. Size Exclusion Chromatography

Prior to gel filtration, 1 mL of ascites was thawed and centrifuged at 2500 g and 4 °C for 10 min to remove cells. Supernatant was taken and mixed 1:2 with TUD buffer (7 M Urea, 2 M Thiourea, 2% (w/v) DTT). For gel filtration, a Superose 6 10/300 column (24 mL bedsize) (GE Healthcare) coupled to an Äkta U-900 FPLC system (GE Healthcare) was applied. Running buffer consisted of 7 M urea, 2 M thiourea, 1 mM DTT. 500 μL of sample was injected and fractions were collected as 500 μL aliquots using a coupled Frac-900 sample fractionator (GE Healthcare).

### 4.5. Dot Blot

From each collected gel filtration fraction 1 μL was spotted on a nitrocellulose membrane (Pall, Port Washington, NY, USA). The membrane was incubated with a directly HRP-conjugated monoclonal anti-CA125 antibody (M11-like, HyTest) at a 1:10.000 dilution and ECL detection (Sigma-Aldrich) was applied.

### 4.6. 1D Electrophoresis

Proteins were precipitated using ReadyPrep 2D clean up kit (Bio-Rad) according to manufacturer’s protocol. Proteins were resuspended in 1% (w/v) SDS. Prior to 1D electrophoresis, 2% (w/v) DTT was added. HiMark prestained (Life Technologies) was used as a molecular mass standard. One-dimensional electrophoresis was carried out on *T* = 3–8% Tris-acetate gels (Life Technologies) according to manufacturers protocol. Proteins were stained with Coomassie brilliant blue overnight.

### 4.7. Tryptic Digest

Bands of interest were excised from the gel manually, destained, reduced with DTT and alkylated with Iodoacetamide (IAA). Digestion was carried out overnight at 37 °C with 100 ng of sequencing grade modified trypsin (Promega).

### 4.8. Filter Aided Digest

Protein concentration of fractions 13–17 derived from size exclusion chromatography were estimated using an EZQ assay (Life Technologies). Filter aided digest was carried out as described in [27]. Deviating from the protocol, 8 M Urea was substituted by 6 M guanidine-hydrochloride, 0.1 M Tris-HCl, 5 mM EDTA, pH 8.0, Lys-C by trypsin (Promega, Madison, WI, USA) and 0.5 M NaCl by 40 mM ammonium bicarbonate. Sample was evaporated in an Alpha 2–4 LD plus vacuum freeze-drier (Martin Christ Gefriertrocknungsanlagen GmbH, Osterode am Harz, Germany) and the peptides were resuspended in FA2 (2% acetonitrile, 0.1% formic acid and 97.9% water).

### 4.9. Mass Spectrometry (HCT Ultra 3D ION Trap)

Mass spectrometry was carried out on an 1100 series HPLC system (Agilent Technologies, Inc., Santa Clara, CA, USA) coupled to a HCT Ultra 3D-Ion-Trap mass spectrometer (Bruker Daltonik GmbH, Bremen, Germany) as described previously [28].

### 4.10. Mass Spectrometry (AmaZon 3D ION Trap)

Mass spectrometry was performed using an online Prominance Nano HPLC system (Shimadzu, Nakagyo-ku, Kyoto, Japan) and amaZon 3D-Ion trap mass spectrometer (Bruker). The LC system was interfaced to the MS using an ESI nano Sprayer (Bruker). 5 μL of sample was loaded on the μ-Precolumn (Thermo Scientific) (Acclaim PepMap100 C18, 5 μm, 100Å, 300 μm inner diameter. ×5 mm) at a flow rate of 5 μL/min in Mobile Phase A (0.1% formic acid (FA) in H_2_O) and resolved on a 75 μm inner diameter × 15 cm, Acclaim PepMap100, C18, 3 μm, 100Å (Thermo Scientific) using a 0–67% gradient of Mobile Phase B (0.1% FA in 90% w/v acetonitrile (ACN)) over 30 min at 400 nL/min (0–60% ACN over 30 min). Ionizable species (300 < *m*/*z* < 1500) were trapped and the three most intense ions eluting at the time were fragmented by collision-induced dissociation. Active exclusion was used to exclude a precursor ion for 0.2 min following the acquisition of two spectra.

### 4.11. Mass Spectrometry (LTQ Orbitrap XL)

Peptides were separated on a HPLC system (Thermo Scientific) using a separation column (Thermo Scientific) (Acclaim PepMap RSLC, C18, pore size 100 Å, particle size 2 μm, 75 μm inner diameter (ID) × 15 cm length) and a trapping column (Thermo Scientific) (Acclaim PepMap100, C18, pore size 100 Å, particle size 3 μm, 75 μm ID ⊕ × 2 cm length). HPLC system was coupled to a LTQ Orbitrap XL mass spectrometer (Thermo Scientific), using the following buffer system: (A) 2% ACN, 0.1% FA in water; (B) 80% ACN, 0.1% FA in water. For in-line desalting and concentration, 2 μL of digest was loaded onto the trap column and then washed for 5 min with 100% A at 5 μL/min flow rate. Peptides were eluted at 300 nL/min flow rate with the following 120 min gradient: 4% B for 10 min, gradient to 55% B over 80 min, gradient to 90% B in 30 s, 90% B for 9.5 min, gradient from 90% to 4% B in 30 s, 4% B for 19.5 min. Full scan mass spectra were acquired in the Orbitrap over *m*/*z* 300–2000. The ten most intense ions at a threshold above 1000 were selected for collision-induced fragmentation in the linear ion trap at normalized collision energy of 35% after accumulation to a target value of 1000. Dynamic exclusion was enabled with a repeat count of 1 and an exclusion mass width by mass 1.50 below and above the precursor ion *m*/*z*. The same precursor was excluded for 30 s.

### 4.12. Data Analysis (HCT Ultra 3D ION Trap and AmaZon 3D ION Trap)

MS and MS/MS spectra were subjected to peak detection and de-convolution using DataAnalysis (Version 3.4; Bruker: Bremen, Germany, 2006). Compound lists were exported into BioTools (Version 3.1; Bruker: Bremen, Germany, 2007) then submitted to MASCOT (Version 2.2; Matrix Science, Inc.: Boston, MA, USA, 2007). Peak list were searched against the Swiss Prot 2011_01 database, mammalian taxonomy. Carbamidomethylation of cysteine was set as a fixed modification and oxidation of methionine was set as variable modification. Peptide tolerance was allowed to be ±0.3 Da, fragment mass tolerance was allowed to be ±0.4 Da, with a maximum of 2 missed cleavages.

### 4.13. Data Analysis (LTQ Orbitrap XL)

Raw data files were subjected to the Proteome Discoverer software (Thermo Scientific) to set up the workflow, files were then submitted to MASCOT (Version 2.2; Matrix Science, Inc.: Boston, MA, USA, 2007) by the Proteome Discoverer Daemon (Thermo Scientific). Peak lists in the range from 350 *m*/*z* to 5000 *m*/*z* were searched against the Swiss Prot 2011_08 database, mammalian taxonomy and the option to match against a MASCOT derived decoy database was set. Carbamidomethylation of cysteine was set as a fixed modification and oxidation of methionine was set as variable modification. Peptide tolerance was allowed to be ±10 ppm; fragment mass tolerance was allowed to be ±0.6 Da, with a maximum of 2 missed cleavages.

### 4.14. First Generation Elisa

ELISA kit for the detection of CA125 levels in human serum was obtained from Abnova (Taipei City, Taiwan) (Lot No. RN-41425) and used according to the manufacturer’s protocol. P95 serum samples were diluted 1:10 and 1:20 in 0.9% (w/v) NaCl in water, fibronectin (MP Biomedicals, Solon, OH, USA) was resuspended in 0.9% (w/v) NaCl in water and 80 μg fibronectin were spiked into P95 serum to obtain a dilution of 1:20. Unspiked 1:10 and 1:20 diluted P95 serum was measured in triplicates, spiked 1:20 diluted P95 serum was measured in sextuplicate for ELISA. The plate was scanned in a Biotrack II plate reader (GE Healthcare) at 450 nm. Standard curve was obtained by plotting absorbance of the manufacturer’s standards against their CA125 concentration (Figure 3(A,B)). To test for statistically significant differences in CA125 readings, the data obtained for the 1:20 diluted P95 serum was compared to the spiked 1:20 diluted P95 serum by a Mann-Whitney *U* test [29] (Figure 3(B,C)).

### 4.15. Second Generation Elisa

Apo-serotransferrin (Merck) and holo-serotransferrin (Merck) were resuspended in H_2_O to a concentration of 100 mg/mL and 100 μL were mixed with 500 μL serum of P607. As a control, 100 μL H_2_O was added to 500 μL of P607 serum. All three samples were given to the IMVS to measure the CA125 concentration. The samples were measured in quintuplicate and tested for statistically significant differences by a Mann-Whitney U test [29] (Figure 3D).

## 5. Conclusions

The results of this study suggest that experimental findings involving the identification and quantification of CA125 should be viewed with caution when they are based on antibody probing alone and have not been verified by detailed mass spectrometric analysis. It is likely that proteins giving false-positive signals are the reason for the conflicting data on the molecular structure of CA125. Furthermore our results show that care should be taken for diagnostics, as the clinical determination of CA125 levels relies exclusively on ELISAs. As a way to overcome potential interferences of CA125 ELISAs with other proteins, an MRM mass spectrometric approach could be applied [30]. Our mass spectrometry results suggest that an assay based on SEC followed by an MRM quantification of several proteotypic peptides of CA125, using triple-quadrupole mass spectrometry instruments and isotopic labeled peptides as internal standards, is achievable.

## Supplementary Materials



## Figures and Tables

**Table 1:** MASCOT search results for peptides of CA125 from SDS-PAGE gel band IV (Figure 1B) identified by a HCT Ultra 3D Ion Trap mass spectrometer. The score of the peptides written in bold exceed the significance threshold (Expectation ≤ 0.05).

Protein name:			Mucin-16			
Mass [Da]:			2359682			
Score:			577			
Matches:			33(5)			
Sequences:			21(5)			
Sequence Coverage: UniProt Acc.:			1% MUC16_HUMAN			

Observed a	z b	Mr (calc) c	Delta d	Score e	Expect f	Peptide
**654.42**	**2**	**1306.6418**	**0.1836**	**84**	**6.80E-07**	**R.NSLYVNGFTHR.S**
**722.45**	**2**	**1442.6572**	**0.2282**	**68**	**2.70E-05**	**K.DGAATGVDAICTHR.L**
**875.05**	**2**	**1747.8628**	**0.2226**	**52**	**0.001**	**K.LTNDIEELGPYTLDR.N**
**655.92**	**2**	**1309.6085**	**0.2170**	**44**	**0.0069**	**K.NTSVGPLYSGCR.L**
**779.93**	**2**	**1557.7206**	**0.1249**	**41**	**0.014**	**K.QEAATGVDTICTHR.V**
654.90	2	1307.6259	0.1596	35	0.06	R.DSLYVNGFTHR.S
625.91	2	1249.6204	0.1851	34	0.076	R.GSLYVNGFTHR.T
485.32	2	968.6018	0.0236	32	0.11	R.LTLLRPEK.D
485.32	2	968.6018	0.0236	32	0.12	R.LTLLRPEK.D
485.35	2	968.6018	0.0836	31	0.12	R.LTLLRPEK.D
555.37	2	1108.5877	0.1378	31	0.14	R.LDPLNPGLDR.E
875.00	2	1747.8628	0.1226	29	0.22	K.LTNDIEELGPYTLDR.N
532.38	2	1062.5346	0.2109	28	0.32	K.ELGPYTLDR.N
532.38	2	1062.5346	0.2109	28	0.34	K.ELGPYTLDR.N
642.40	2	1282.5976	0.1879	26	0.46	K.STSVGPLYSGCR.L
598.41	2	1194.5921	0.2134	25	0.49	R.EQLYWELSK.L
596.67	3	1786.8268	0.1614	25	0.51	R.SEKDGAATGVDAICTHR.L
642.40	2	1282.5976	0.1879	25	0.57	K.STSVGPLYSGCR.L
485.39	2	968.6018	0.1636	22	0.7	R.LTLLRPEK.D
399.85	2	797.5123	0.1731	22	0.73	R.VLQGLLR.S
654.90	2	1307.6259	0.1596	24	0.81	R.DSLYVNGFTHR.S
679.95	2	1357.7605	0.1249	23	1	R.GIIELGPYLLDR.G
570.42	2	1138.6022	0.2232	20	1.3	R.VAIYEEFLR.M
662.46	2	1322.7082	0.1973	20	1.3	R.DIQDKVTTLYK.G
532.33	2	1062.5346	0.1109	22	1.4	K.ELGPYTLDR.N
598.41	2	1194.5921	0.2134	21	1.4	R.EQLYWELSK.L
436.95	3	1307.6259	0.2023	20	1.8	R.DSLYVNGFTHR.S
875.00	2	1747.8628	0.1226	20	1.9	K.LTNDIEELGPYTLDR.N
594.27	3	1779.9016	−0.1135	15	5.5	K.QVFHELSQQTHGITR.L
663.40	2	1324.6735	0.1119	11	13	R.LDPTGPGLDRER.L
628.43	2	1254.8064	0.0391	10	18	R.VLQGLLKPLFK.S

a Observed mass/charge of the peptide in Da;

b Charge state of the peptide.

c Theoretical peptide mass in Da;

d difference between the observed and the theoretical mass in Da;

e MASCOT score;

f Expectation of a random match, derived from the MASCOT score;

g Mass accuracy in parts per million.

**Table 2:** Combined MASCOT search results for SEC fractions 15–17 in which CA125 was identified by a LTQ Orbitrap XL mass spectrometer. The score of the peptides written in bold exceed the significance threshold (Expectation ≤ 0.05).

Observed a	z b	Mr (calc) c	ppm d	Score e	Expect f	Peptide
**748.8278**	**2**	**1495.64**	**0.60**	**52**	**4.60E–05**	**K.SYFSDCQVSTFR.S**
**798.7573**	**3**	**2393.249**	**0.60**	**56**	**5.90E–05**	**R.LTLLRPEKDGAATGVDAICTHR.L**
**570.3081**	**2**	**1138.602**	−**0.50**	**44**	**0.0008**	**R.VAIYEEFLR.M**
**399.7638**	**2**	**797.5123**	**1.05**	**37**	**0.00099**	**R.VLQGLLR.S**
**655.812**	**2**	**1309.609**	**0.75**	**42**	**0.0011**	**K.NTSVGPLYSGCR.L**
**721.3632**	**2**	**1440.714**	−**1.80**	**39**	**0.003**	**K.HGAATGVDAICTLR.L**
**642.3064**	**2**	**1282.598**	**0.51**	**36**	**0.0039**	**K.STSVGPLYSGCR.L**
**596.814**	**4**	**2383.228**	−**0.32**	**38**	**0.0047**	**R.LTLLRSEKDGAATGVDAICTHR.L**
**654.3267**	**2**	**1306.642**	−**2.25**	**37**	**0.0058**	**R.NSLYVNGFTHR.S**
**601.8525**	**2**	**1201.689**	**1.02**	**34**	**0.0078**	**R.VLQGLLGPMFK.N**
**523.6422**	**3**	**1567.905**	**0.03**	**30**	**0.011**	**R.LTLLRPEKDGVATR.V**
**607.8801**	**2**	**1213.743**	**1.87**	**27**	**0.014**	**R.VLQGLLSPIFK.N**
**837.1103**	**3**	**2508.312**	−**1.12**	**30**	**0.022**	**R.LTLLRPEKQEAATGVDTICTHR.V**
**677.8256**	**2**	**1353.635**	**1.48**	**28**	**0.037**	**K.NTSIGPLYSSCR.L**
**798.1088**	**3**	**2391.306**	−**0.49**	**25**	**0.04**	**R.LTLLRPEKHGAATGVDAICTLR.L**
**594.3086**	**3**	**1779.902**	**1.29**	**28**	**0.049**	**K.QVFHELSQQTHGITR.L**
711.1059	4	2840.395	−0.15	27	0.053	R.LTSLRPEKDGAATGMDAVCLYHPNPK.R
679.8878	2	1357.761	0.41	25	0.077	R.GIIELGPYLLDR.G
628.4118	2	1254.806	2.14	12	0.086	R.VLQGLLKPLFK.S
1007.534	2	2013.053	0.59	26	0.088	K.LSQLTHGITELGPYTLDR.H
947.8076	3	2840.395	2.15	25	0.089	R.LTSLRPEKDGAATGMDAVCLYHPNPK.R
514.2987	3	1539.873	0.68	16	0.43	R.LTLLRPEKDGAATR.V
717.8562	2	1433.697	0.39	17	0.57	K.VDAICTYRPDPK.S
598.3046	2	1194.592	2.12	15	0.61	R.EQLYWELSK.L
607.3171	3	1818.926	1.65	17	0.69	K.SPGLNREQLYWELSK.L
555.9742	3	1664.9	0.54	13	1.2	R.RVDRVAIYEEFLR.M
514.2987	3	1539.873	0.68	10	1.8	R.LTLLRPEKDGAATR.V
477.5088	4	1906.01	−1.85	6	6.3	K.NTSVGPLYSGCRLTLLR.S
862.958	2	1723.903	−1.02	5	7.8	K.DPEILSWTIPPSIEK.T
647.5736	4	2586.261	1.71	5	8.8	K.HEAATGVDTICTHRVDPIGPGLDR.E
702.6927	3	2105.058	−0.61	4	11	K.KDGAATKVDAICTYRPDPK.S
848.0892	3	2541.237	3.53	1	19	K.SKLSLTPGLMETSISEETSSATEK.S

a Observed mass/charge of the peptide in Da;

b Charge state of the peptide;

c Theoretical peptide mass in Da;

d Mass accuracy in parts per million;

e MASCOT score;

f Expectation of a random match, derived from the MASCOT score.
