# Supplementary material for: Methods for Identification of CA125 from Ovarian Cancer Ascites by High Resolution Mass Spectrometry
Source: Int J Mol Sci. 2012 Aug 9;13(8):9942–58. doi: 10.3390/ijms13089942 (PMC3431838; doi:10.3390/ijms13089942)
Supplement: Supplementary file 1 [file ijms-13-09942-s001.pdf]

Methods for Identification of CA125 from Ovarian Cancer Ascites by High Resolution Mass Spectrometry

Supplementary Information

Figure S1. (**A**) 1D (T = 3–8%) western-blot of P517 ascites probed with M11-like antibody. Numbers from 1–7 indicate positive signals, M indicates the molecular mass marker lane; (**B**) Coomassie brilliant blue stained proteins from P517 ascites (T = 3–8%). Roman numbers indicate cut-out bands corresponding to positive signals in the western-blot. These bands were subjected to mass-spectrometry (see Table S1).

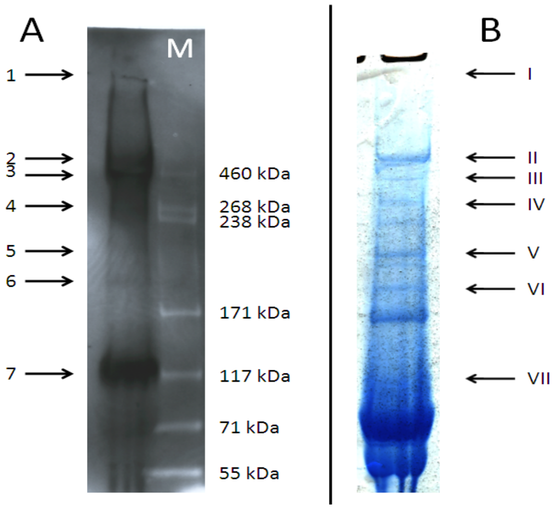

Table S1. Identified proteins from 1D SDS-PAGE bands showing positive signal with M11-like antibody (see also Figure S1).

Band	Protein name	Mass [Da]	Seq. coverage	Peptides	Score	Uniprot Acc.
I	Apolipoprotein B-100	516,651	18%	78(20)	2,217	APOB_HUMAN
I	Inter-alpha-trypsin inhibitor heavy chain H1	101,782	9%	9(4)	336	ITIH1_HUMAN
I	Serum albumin	71,317	9%	7(3)	195	ALBU_HUMAN
I	Ig gamma-1 chain C region	36,596	12%	5(2)	140	IGHG1_HUMAN
II	Apolipoprotein B-100	516,651	38%	183(104)	7,763	APOB_HUMAN
II	Serum albumin	71,317	36%	25(16)	1,078	ALBU_HUMAN
II	Ig gamma-1 chain C region	36,596	16%	5(4)	231	IGHG1_HUMAN
II	Alpha-2-macroglobulin	164,613	3%	3(2)	121	A2MG_HUMAN
III	Apolipoprotein B-100	516,651	23%	97(58)	3,961	APOB_HUMAN
III	Serum albumin	71,317	36%	29(17)	1,133	ALBU_HUMAN
III	Alpha-2-macroglobulin	164,613	10%	13(5)	481	A2MG_HUMAN
III	Fibronectin	266,052	6%	10(4)	417	FINC_HUMAN
III	Ig gamma-1 chain C region	36,596	19%	8(5)	257	IGHG1_HUMAN
III	Inter-alpha-trypsin inhibitor heavy chain H1	101,782	7%	4(3)	172	ITIH1_HUMAN
III	Complement C3	188,569	1%	2(2)	154	CO3_HUMAN
III	Inter-alpha-trypsin inhibitor heavy chain H2	106,853	5%	3(2)	123	ITIH2_HUMAN

Table S1. *Cont.*

Band	Protein name	Mass [Da]	Seq. coverage	Peptides	Score	Uniprot Acc.
III	Ig kappa chain C region	11,773	32%	4(2)	117	IGKC_HUMAN
IV	Apolipoprotein B-100	516,651	7%	36(9)	989	APOB_HUMAN *
IV	Fibronectin	266,052	11%	24(9)	783	FINC_HUMAN *
IV	Serum albumin	71,317	21%	16(10)	682	ALBU_HUMAN *
IV	Alpha-2-macroglobulin	164,613	8%	13(5)	460	A2MG_HUMAN *
IV	Inter-alpha-trypsin inhibitor heavy chain H1	101,782	6%	5(3)	202	ITIH1_HUMAN *
IV	Inter-alpha-trypsin inhibitor heavy chain H2	106,853	10%	9(2)	187	ITIH2_HUMAN *
IV	Ig gamma-1 chain C region	36,596	23%	7(2)	186	IGHG1_HUMAN *
V	Alpha-2-macroglobulin	164,613	40%	84(70)	2,972	A2MG_HUMAN
V	Serum albumin	71,317	45%	36(24)	1,441	ALBU_HUMAN
V	Pregnancy zone protein	165,242	9%	16(13)	595	PZP_HUMAN
V	Complement factor H	143,680	11%	11(8)	592	CFAH_HUMAN
V	Ig gamma-1 chain C	36,596	23%	9(6)	301	IGHG1_HUMAN
V	Complement C3	188,569	3%	5(3)	266	CO3_HUMAN
V	Ig gamma-2 chain C	36,505	22%	7(5)	235	IGHG2_HUMAN
V	Ig mu chain C region	49,960	7%	3(2)	168	IGHM_HUMAN
V	Ig kappa chain C region	11,773	32%	4(3)	126	IGKC_HUMAN
VI	Ceruloplasmin	122,983	36%	35(22)	1,406	CERU_HUMAN
VI	Serum albumin	71,317	31%	30(17)	1,195	ALBU_HUMAN
VI	Complement C3	188,569	8%	13(4)	464	CO3_HUMAN
VI	Alpha-2-macroglobulin	164,613	8%	9(2)	247	A2MG_HUMAN
VI	Ig gamma-1 chain C	36,596	19%	9(5)	234	IGHG1_HUMAN
VI	Ig gamma-2 chain C	36,505	19%	7(3)	207	IGHG2_HUMAN
VI	Serotransferrin	79,294	9%	6(2)	195	TRFE_HUMAN
VI	Ig heavy chain V-III region TIL	12,462	26%	2(2)	119	HV304_HUMAN
VII	Serotransferrin	79,294	52%	42(30)	1,938	TRFE_HUMAN
VII	Serum albumin	71,317	45%	36(28)	1,408	ALBU_HUMAN
VII	Ig gamma-1 chain C	36,596	50%	20(14)	580	IGHG1_HUMAN
VII	Ig mu chain C region	49,960	21%	11(5)	330	IGHM_HUMAN
VII	Ig lambda-2 chain C regions	11,458	85%	7(5)	315	LAC2_HUMAN
VII	Ig gamma-2 chain C region	36,505	28%	10(6)	310	IGHG2_HUMAN
VII	Gelsolin	86,043	8%	4(3)	252	GELS_HUMAN
VII	Alpha-1B-glycoprotein	54,790	15%	6(2)	225	A1BG_HUMAN
VII	Ig kappa chain C region	11,773	50%	9(6)	208	IGKC_HUMAN
VII	Fibrinogen gamma chain	52,106	9%	3(3)	168	FIBG_HUMAN
VII	Ig heavy chain V-III region VH26	12,745	29%	2(2)	166	HV303_HUMAN
VII	Complement C3	188,569	2%	2(2)	163	CO3_HUMAN
VII	Ig kappa chain V-I region	12,099	31%	3(2)	155	KV101_HUMAN
VII	Ig kappa chain V-III region SIE	11,882	24%	2(2)	91	KV302_HUMAN
VII	Alpha-1-antichymotrypsin	47,792	9%	4(2)	82	AACT_HUMAN

Roman numbers in "Band" column correspond to Roman numbers in Supplement Data 1B; Excluded: Trypsin, Keratins, Hits with peptides under significance score only, hits with one significant peptide; Proteins identified by AmaZon 3D ION TRAP; *: Proteins identified by LTQ Orbitrap mass spectrometer.

Figure S2. SEC fractions, 1 µL probed with M11-like antibody.

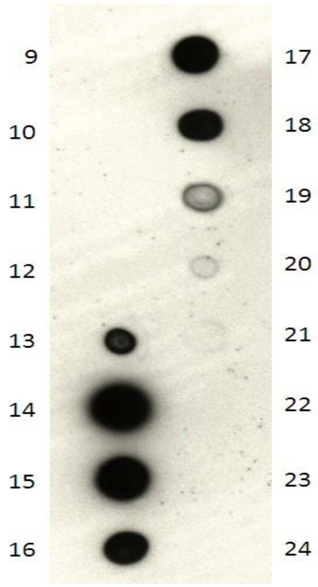

Table S2. Identified proteins from 1D SDS-PAGE bands showing positive signal with M11-like antibody, SEC fractions 14 and 15 (see Figure 1B).

Band	Protein name	Mass [Da]	Seq. coverage	Peptides	Sequences	Score	Uniprot Acc.
I	-	-	-	-	-	-	-
II	Apolipoprotein B-100	516,651	22%	114(70)	83(53)	3,882	APOB_HUMAN
II	Apolipoprotein(a)	514,737	5%	48(35)	19(17)	1,192	APOA_HUMAN
II	Proteoglycan 4	152,238	5%	8(4)	6(4)	269	PRG4_HUMAN
III	Apolipoprotein B-100	516,651	40%	257(207)	141(116)	9,012	APOB_HUMAN
III	Proteoglycan 4	152,238	3%	3(2)	3(2)	140	PRG4_HUMAN
IV	Apolipoprotein B-100	516,651	13%	71(34)	53(27)	1,981	APOB_HUMAN
IV	**Mucin-16**	**2,359,682**	**1%**	**33(5)**	**21(5)**	**577**	**MUC16_HUMAN**
IV	Inter-alpha-trypsin inhibitor heavy chain H1	101,782	7%	8(6)	6(4)	321	ITIH1_HUMAN
IV	Mucin-5B	611,584	1%	6(3)	5(3)	233	MUC5B_HUMAN
IV	Inter-alpha-trypsin inhibitor heavy chain H2	106,853	6%	5(3)	4(3)	198	ITIH2_HUMAN
IV	Complement C3	188,569	4%	6(3)	5(3)	196	CO3_HUMAN

Roman numbers in "Band" column correspond to Roman numbers in Figure 1B; Excluded: Trypsin, Keratins, hits with peptides under significance score only, hits with one significant peptide; Proteins identified by HCT Ultra 3D-Ion-Trap.

Table S3. Proteins identified from 2D gel electrophoresis protein spots giving positive signals with a M11-like antibody (see Figure 2A,C).

Spot number	Protein name	Mass [Da]	pI	Seq. coverage	Peptides	Score	Uniprot Acc.
1	Fibronectin	266,052	5.46	5%	9(3)	225	FINC_HUMAN
2	Fibronectin	266,052	5.46	6%	13(3)	305	FINC_HUMAN
3	Alpha-2-macroglobulin	164,613	6.03	17%	29(11)	762	A2MG_HUMAN
4	Alpha-2-macroglobulin	164,613	6.03	27%	41(16)	1,013	A2MG_HUMAN
4	Complement C3	188,569	6.02	7%	11(2)	215	CO3_HUMAN
5	Fibrinogen gamma chain	52,106	5.37	30%	14(3)	312	FIBG_HUMAN
5	Complement C4-A	194,247	6.65	4%	7(2)	139	CO4A_HUMAN
6	Fibrinogen gamma chain	52,106	5.37	34%	13(3)	333	FIBG_HUMAN
7	Fibrinogen gamma chain	52,106	5.37	15%	6(2)	183	FIBG_HUMAN
8	Serum albumin	71,317	5.92	25%	15(7)	498	ALBU_HUMAN
8	Fibrinogen gamma chain	52,106	5.37	25%	11(7)	350	FIBG_HUMAN
9	Serotransferrin	79,294	6.81	57%	44(30)	1,983	TRFE_HUMAN
9	Ig mu chain C region	49,960	6.35	33%	12(7)	447	IGHM_HUMAN
10	Serotransferrin	79,294	6.81	70%	54(39)	2,778	TRFE_HUMAN
10	Ig mu chain C region	49,960	6.35	15%	5(3)	177	IGHM_HUMAN
11	Serotransferrin	79,294	6.81	58%	45(39)	2,785	TRFE_HUMAN
11	Ig mu chain C region	49,960	6.35	10%	3(3)	168	IGHM_HUMAN
12	Serotransferrin	79,294	6.81	57%	50(37)	2,763	TRFE_HUMAN
12	Ig mu chain C region	49,960	6.35	17%	7(3)	291	IGHM_HUMAN
13	Ig kappa chain C region	11,773	5.58	32%	2(2)	102	IGKC_HUMAN **
14	Fibrinogen beta chain	56,577	8.54	17%	8(2)	168	FIBB_HUMAN
14	Ig gamma-2 chain C region	36,505	7.66	22%	10(2)	161	IGHG2_HUMAN
14	Ig kappa chain V-IV region Len	12,746	7.92	23%	3(2)	88	KV402_HUMAN
15	Alpha-1-antitrypsin	46,878	5.37	9%	3(1)	105	A1AT_HUMAN *
15	Ig kappa chain C region	11,773	5.58	32%	5(3)	89	IGKC_HUMAN *
16	Ig kappa chain C region	11,773	5.58	48%	13(9)	146	IGKC_HUMAN *
17	Ig kappa chain C region	11,773	5.58	32%	10(6)	118	IGKC_HUMAN *
18	Complement C3	188,569	6.02	23%	79(63)	2,812	CO3_HUMAN
19	Ig gamma-1 chain C region	36,596	8.46	35%	19(5)	243	IGHG1_HUMAN
19	Ig gamma-2 chain C region	36,505	7.66	23%	16(6)	237	IGHG2_HUMAN
20	Ig kappa chain C region	11,773	5.58	32%	22(11)	129	IGKC_HUMAN *
21	Ig kappa chain C region	11,773	5.58	32%	7(4)	112	IGKC_HUMAN *
22	Ig gamma-1 chain C	36,596	8.46	35%	18(7)	339	IGHG1_HUMAN
22	Ig kappa chain C region	11,773	5.58	64%	10(3)	214	IGKC_HUMAN
23	Ig kappa chain C region	11,773	5.58	64%	13(7)	305	IGKC_HUMAN
23	Ig gamma-1 chain C region	36,596	8.46	31%	16(5)	303	IGHG1_HUMAN
23	Ig kappa chain V-III region SIE	11,882	8.70	39%	4(2)	133	KV302_HUMAN
24	Ig kappa chain C region	11,773	5.58	64%	11(4)	209	IGKC_HUMAN
24	Ig gamma-1 chain C region	36,596	8.46	23%	11(3)	183	IGHG1_HUMAN
24	Ig kappa chain V-III region WOL	11,853	9.07	50%	4(2)	180	KV305_HUMAN

Table S3. *Cont.*

Spot number	Protein name	Mass [Da]	pI	Seq. coverage	Peptides	Score	Uniprot Acc.
25	Ig gamma-1 chain C	36,596	8.46	32%	24(15)	399	IGHG1_HUMAN
25	Ig gamma-2 chain C region	36,505	7.66	39%	25(6)	349	IGHG2_HUMAN
25	Ig heavy chain V-III region VH26	12,745	8.49	22%	3(3)	229	HV303_HUMAN
26	Pigment epithelium-derived factor	46,454	5.97	14%	9(3)	186	PEDF_HUMAN
27	Fibrinogen gamma chain	52,106	5.37	48%	25(7)	498	FIBG_HUMAN
27	Pigment epithelium-derived factor	46,454	5.97	14%	7(2)	137	PEDF_HUMAN
28	Fibrinogen gamma chain	52,106	5.37	62%	28(13)	808	FIBG_HUMAN
28	Transthyretin	15,991	5.52	68%	11(7)	402	TTHY_HUMAN
28	Pigment epithelium-derived factor	46,454	5.97	25%	9(4)	328	PEDF_HUMAN
29	Fibrinogen beta chain	56,577	8.54	53%	36(11)	754	FIBB_HUMAN
29	Haptoglobin	45,861	6.13	7%	4(2)	127	HPT_HUMAN
30	Fibrinogen beta chain	56,577	8.54	44%	24(12)	702	FIBB_HUMAN
30	Apolipoprotein L1	44,004	5.60	8%	5(3)	241	APOL1_HUMAN
31	Ig kappa chain C region	11,773	5.58	90%	18(18)	528	IGKC_HUMAN
31	Ig kappa chain V-I region OU	11,884	9.94	16%	4(4)	218	KV114_HUMAN
31	Ig kappa chain V-I region AG	12,099	5.67	31%	6(6)	214	KV101_HUMAN
31	Apolipoprotein A-I	30,759	5.56	20%	5(2)	179	APOA1_HUMAN
31	Serum amyloid P-component	25,485	6.10	11%	3(2)	129	SAMP_HUMAN
32	Ig lambda-2 chain C regions	11,458	6.92	69%	11(4)	188	LAC2_HUMAN
32	Ig kappa chain C region	11,773	5.58	64%	6(2)	117	IGKC_HUMAN
33	Ig kappa chain C region	11,773	5.58	89%	30(23)	457	IGKC_HUMAN
33	Ig kappa chain V-II region TEW	12,422	5.69	41%	9(7)	302	KV204_HUMAN
33	Ig kappa chain V-III region SIE	11,882	8.70	39%	3(3)	137	KV302_HUMAN
33	Ig kappa chain V-IV region Len	12,746	7.92	23%	2(2)	134	KV402_HUMAN
33	Ig lambda-2 chain C regions	11,458	6.92	62%	4(2)	115	LAC2_HUMAN
34	Ig lambda-2 chain C regions	11,458	6.92	69%	13(1)	126	LAC2_HUMAN
35	Ig lambda-1 chain C regions	11,512	7.89	74%	13(4)	162	LAC1_HUMAN
35	Ig kappa chain C region	11,773	5.58	64%	12(2)	112	IGKC_HUMAN
36	Ig kappa chain C region	11,773	5.58	90%	39(34)	592	IGKC_HUMAN
36	Ig kappa chain V-I region DEE	11,768	9.43	22%	4(4)	286	KV105_HUMAN
36	Ig kappa chain V-IV region Len	12,746	7.92	36%	13(12)	242	KV402_HUMAN
36	Ig kappa chain V-III region SIE	11,882	8.70	39%	5(5)	226	KV302_HUMAN
36	Ig lambda-2 chain C regions	11,458	6.92	62%	9(5)	172	LAC2_HUMAN
37	Ig kappa chain C region	11,773	5.58	67%	165(146)	323	IGKC_HUMAN *
37	Ig kappa chain V-II region TEW	12,422	5.69	38%	10(7)	228	KV204_HUMAN *
37	Ig kappa chain V-III region SIE	11,882	8.70	39%	10(6)	198	KV302_HUMAN *
37	Ig kappa chain V-IV region Len	12,746	7.92	36%	6(5)	176	KV402_HUMAN *
38	Ig kappa chain C region	11,773	5.58	85%	39(32)	535	IGKC_HUMAN
38	Ig kappa chain V-III region SIE	11,882	8.70	45%	13(9)	315	KV302_HUMAN
38	Ig kappa chain V-I region DEE	11,768	9.43	22%	4(3)	225	KV105_HUMAN
38	Ig kappa chain V-IV region Len	12,746	7.92	36%	4(4)	196	KV402_HUMAN
38	Ig kappa chain V-III region B6	11,742	9.34	16%	8(2)	144	KV301_HUMAN
38	Ig kappa chain V-II region TEW	12,422	5.69	32%	3(3)	128	KV204_HUMAN
38	Ig kappa chain V-III region VG (Fragment)	12,681	4.85	26%	4(2)	93	KV309_HUMAN

Table S3. *Cont.*

Spot number	Protein name	Mass [Da]	pI	Seq. coverage	Peptides	Score	Uniprot Acc.
39	Ig lambda-1 chain C regions	11,512	7.89	69%	15(11)	270	LAC1_HUMAN
39	Ig lambda-2 chain C regions	11,458	6.92	69%	15(10)	266	LAC2_HUMAN
40	Ig kappa chain C region	11,773	5.58	89%	31(24)	539	IGKC_HUMAN
40	Ig kappa chain V-III region WOL	11,853	9.07	50%	10(9)	300	KV305_HUMAN
40	Ig kappa chain V-I region DEE	11,768	9.43	31%	5(4)	204	KV105_HUMAN
40	Ig kappa chain V-III region B6	11,742	9.34	31%	7(4)	189	KV301_HUMAN
40	Ig kappa chain V-I region EU	11,895	8.62	32%	4(2)	158	KV106_HUMAN
40	Ig kappa chain V-IV region Len	12,746	7.92	23%	2(2)	114	KV402_HUMAN

Spot numbers corresponding to Figure 2C; Excluded: Trypsin, Keratins, Hits with peptides under significance score only, hits with one significant peptide; Proteins identified by HCT Ultra 3D-Ion-Trap; * Proteins identified by AmaZon 3D ION TRAP; ** 5µL injection volume, no protein identification with 2µL injection volume.

Table S4. Proteins identified from 2D gel electrophoresis protein spots giving positive signals with an OC125-like antibody (see Figure 2B,D).

Spot number	Protein name	Mass [Da]	pI	Seq. coverage	Peptides	Score	Uniprot Acc.
1	Alpha-2-macroglobulin	164,613	6.03	8%	10(8)	443	A2MG_HUMAN *
2	Alpha-2-macroglobulin	164,613	6.03	11%	18(14)	659	A2MG_HUMAN
2	Serum albumin	71,317	5.92	7%	4(2)	164	ALBU_HUMAN
2	Ig kappa chain C region	11,773	5.58	32%	20(9)	145	IGKC_HUMAN
3	Alpha-2-macroglobulin	164,613	6.03	3%	7(5)	200	A2MG_HUMAN
3	Serum albumin	71,317	5.92	6%	4(4)	173	ALBU_HUMAN
4	Complement C3	188,569	6.02	2%	6(3)	173	CO3_HUMAN
4	Ig kappa chain C region	11,773	5.58	32%	4(3)	78	IGKC_HUMAN
5	Complement C3	188,569	6.02	23%	70(51)	1587	CO3_HUMAN
5	Serum albumin	71,317	5.92	7%	5(3)	196	ALBU_HUMAN
5	Ig kappa chain V-IV region Len	12,746	7.92	36%	8(4)	194	KV402_HUMAN
5	Ig kappa chain C region	11,773	5.58	48%	143(116)	168	IGKC_HUMAN
6	Ig kappa chain C region	11,773	5.58	61%	103(59)	213	IGKC_HUMAN
6	Complement C3	188,569	6.02	3%	7(2)	150	CO3_HUMAN
6	Ig kappa chain V-IV region Len	12,746	7.92	23%	2(2)	101	KV402_HUMAN
6	Serum albumin	71,317	5.92	3%	2(2)	97	ALBU_HUMAN
7	Complement C3	188,569	6.02	7%	8(5)	377	CO3_HUMAN *
8	Serum albumin	71,317	5.92	9%	6(5)	368	ALBU_HUMAN *
9	Serum albumin	71,317	5.92	7%	5(4)	354	ALBU_HUMAN *
9	Ig kappa chain C region	11,773	5.58	32%	2(2)	77	IGKC_HUMAN *
10	Serum albumin	71,317	5.92	9%	12(8)	313	ALBU_HUMAN
10	Complement C4-A	194,247	6.65	2%	3(2)	120	CO4A_HUMAN
10	Complement C4-B	194,212	6.73	2%	3(2)	120	CO4B_HUMAN
10	Ig kappa chain C region	11,773	5.58	32%	2(2)	111	IGKC_HUMAN
11	Alpha-1-antitrypsin	46,878	5.37	36%	62(40)	809	A1AT_HUMAN
11	Angiotensinogen	53,406	5.87	14%	7(6)	352	ANGT_HUMAN
11	Antithrombin-III	53,025	6.32	10%	7(7)	224	ANT3_HUMAN

Table S4. *Cont.*

Spot number	Protein name	Mass [Da]	pI	Seq. coverage	Peptides	Score	Uniprot Acc.
11	Vitamin D-binding protein	54,526	5.40	10%	11(4)	213	VTDB_HUMAN
11	Ig kappa chain C region	11,773	5.58	32%	5(3)	85	IGKC_HUMAN
12	Fibrinogen beta chain	56,577	8.54	42%	70(23)	1,003	FIBB_HUMAN
12	Serum albumin	71,317	5.92	8%	10(9)	300	ALBU_HUMAN
12	Ig kappa chain C region	11,773	5.58	32%	34(13)	138	IGKC_HUMAN
13	No identification	-	-	-	-	-	- *
14	No identification	-	-	-	-	-	- *
15	No identification	-	-	-	-	-	- *

Spot numbers correspond to Figure 2D; Excluded: Trypsin, Keratins, Hits with peptides under significance score only, hits with one significant peptide; Proteins identified by AmaZon 3D ION TRAP; *: 5μL injection volume, no identification with 2μL injection volume.

Figure S3. (**a**) MS/MS spectra and by MASCOT assigned fragment ions. CA125 peptide: K.SYFSDCQVSTFR.S; Rank in Table 2: 1; (**b**) MS/MS spectra and by MASCOT assigned fragment ions; CA125 peptide:R.LTLLRPEKDGAATGVDAICTHR.L; Rank in Table 2: 2; (**c**) MS/MS spectra and by MASCOT assigned fragment ions; CA125 peptide: R.VAIYEEFLR.M; Rank in Table 2: 3; (**d**) MS/MS spectra and by MASCOT assigned fragment ions; CA125 peptide: R.VLQGLLR.S; Rank in Table 2: 4; (**e**) MS/MS spectra and by MASCOT assigned fragment ions; CA125 peptide: K.NTSVGPLYSGCR.L; Rank in Table 2: 5; (**f**) MS/MS spectra and by MASCOT assigned fragment ions; CA125 peptide: K.HGAATGVDAICTLR.L; Rank in Table 2: 6; (**g**) MS/MS spectra and by MASCOT assigned fragment ions; CA125 peptide: K.STSVGPLYSGCR.L; Rank in Table 2: 7; (**h**) MS/MS spectra and by MASCOT assigned fragment ions; CA125 peptide: R.LTLLRSEKDGAATGVDAICTHR.L; Rank in Table 2: 8; (**i**) MS/MS spectra and by MASCOT assigned fragment ions; CA125 peptide: R.NSLYVNGFTHR.S; Rank in Table 2: 9; (**j**) MS/MS spectra and by MASCOT assigned fragment ions; CA125 peptide: R.VLQGLLGPMFK.N; Rank in Table 2: 10; (**k**) MS/MS spectra and by MASCOT assigned fragment ions; CA125 peptide: R.LTLLRPEKDGVATR.V; Rank in Table 2: 11; (**l**) MS/MS spectra and by MASCOT assigned fragment ions; CA125 peptide: R.VLQGLLSPIFK.N; Rank in Table 2: 12; (**m**) MS/MS spectra and by MASCOT assigned fragment ions; CA125 peptide: R.LTLLRPEKQEAATGVDTICTHR.V; Rank in Table 2: 13; (**n**) MS/MS spectra and by MASCOT assigned fragment ions; CA125 peptide: K.NTSIGPLYSSCR.L; Rank in Table 2: 14; (**o**) MS/MS spectra and by MASCOT assigned fragment ions; CA125 peptide: R.LTLLRPEKHGAATGVDAICTLR.L; Rank in Table 2: 15; (**p**) MS/MS spectra and by MASCOT assigned fragment ions; CA125 peptide: K.QVFHELSQQTHGITR.L; Rank in Table 2: 16.

(a)

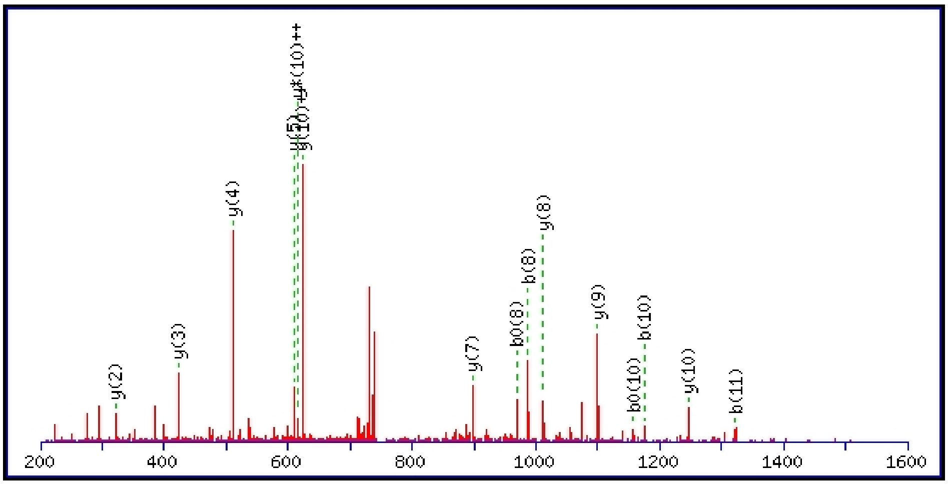
y(2)
y(3)
y(4)
y(5)
y(10)++
y*(10)++
y(7)
b0(8)
b(8)
y(8)
y(9)
b0(10)
b(10)
y(10)
b(11)
200
400
600
800
1000
1200
1400
1600

(b)

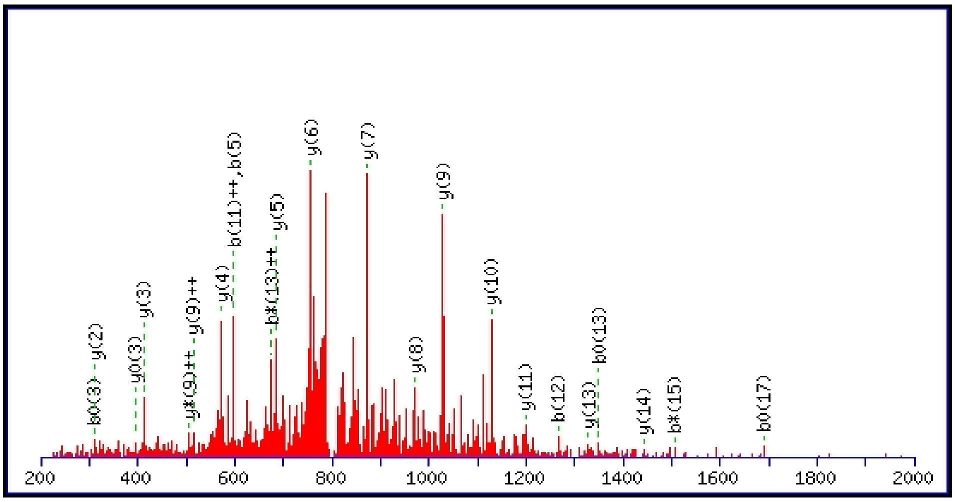
b0(3)
y(2)
y0(3)
y(3)
y*(9)++
y(9)++
y(4)
b(11)++,b(5)
b*(13)++
y(5)
y(6)
y(7)
y(8)
y(9)
y(10)
y(11)
b(12)
y(13)
b0(13)
y(14)
b*(15)
b0(17)
200
400
600
800
1000
1200
1400
1600
1800
2000

(c)

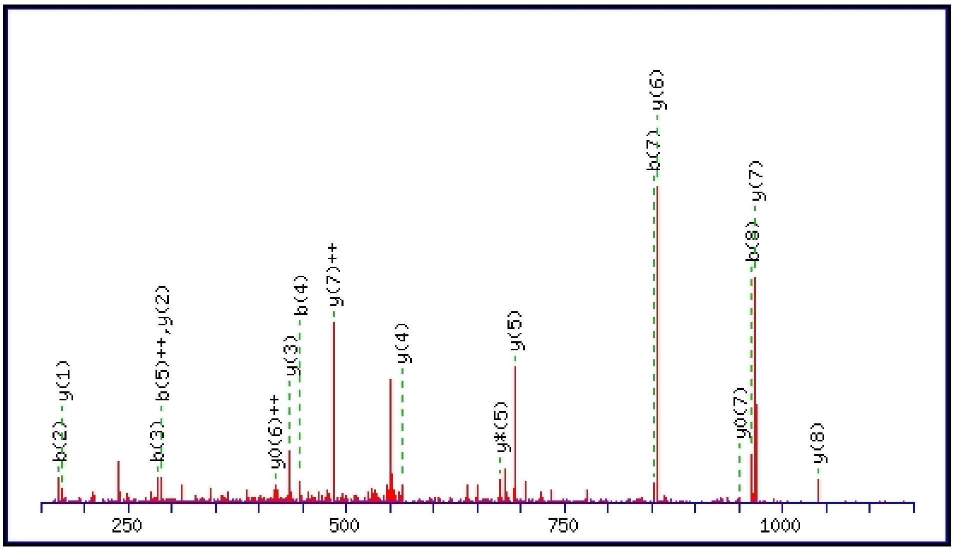
b(2)
y(1)
b(3)
b(5)++,y(2)
y0(6)++
y(3)
b(4)
y(7)++
y(4)
y*(5)
y(5)
b(7)
y(6)
y0(7)
b(8)
y(7)
y(8)
250
500
750
1000

(d)

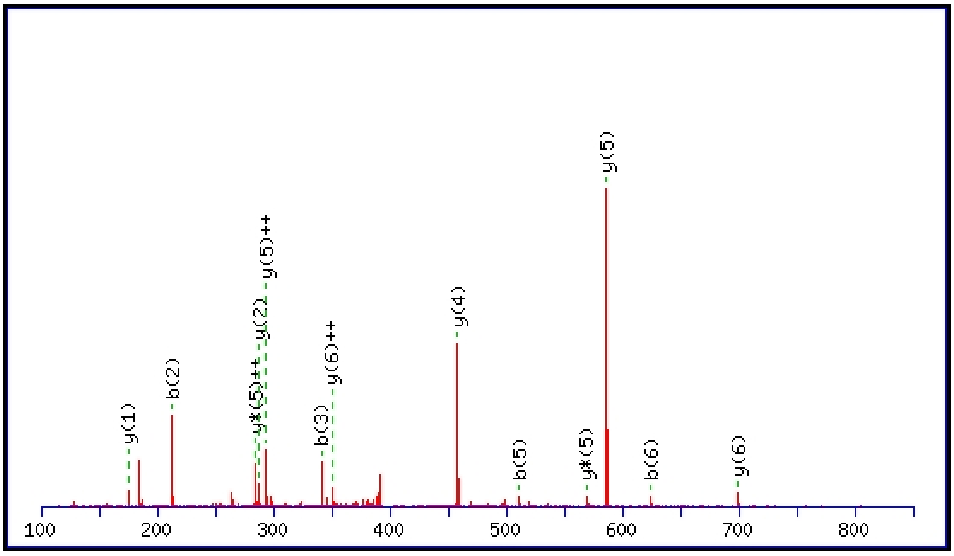
y(1)
b(2)
y*(5)++
y(2)
y(5)++
b(3)
y(6)++
y(4)
b(5)
y*(5)
y(5)
b(6)
y(6)
100
200
300
400
500
600
700
800

(e)

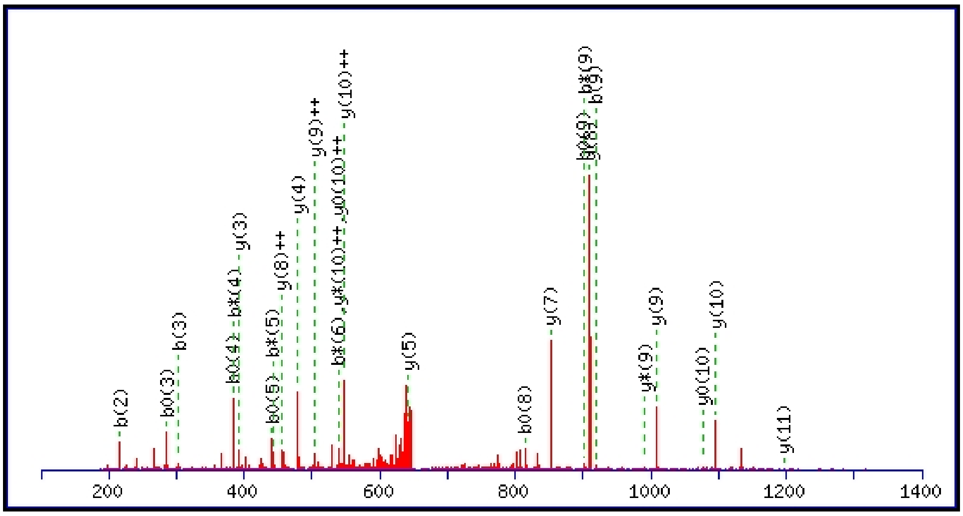
b(2)
b0(3)
b(3)
b0(4)
b*(4)
y(3)
b0(5)
b*(5)
y(8)++
y(4)
y(9)++
b*(6)
y*(10)++,y0(10)++
y(10)++
y(5)
b0(8)
y(7)
y*(9)
y(9)
y0(10)
y(10)
y(11)
200
400
600
800
1000
1200
1400

(f)

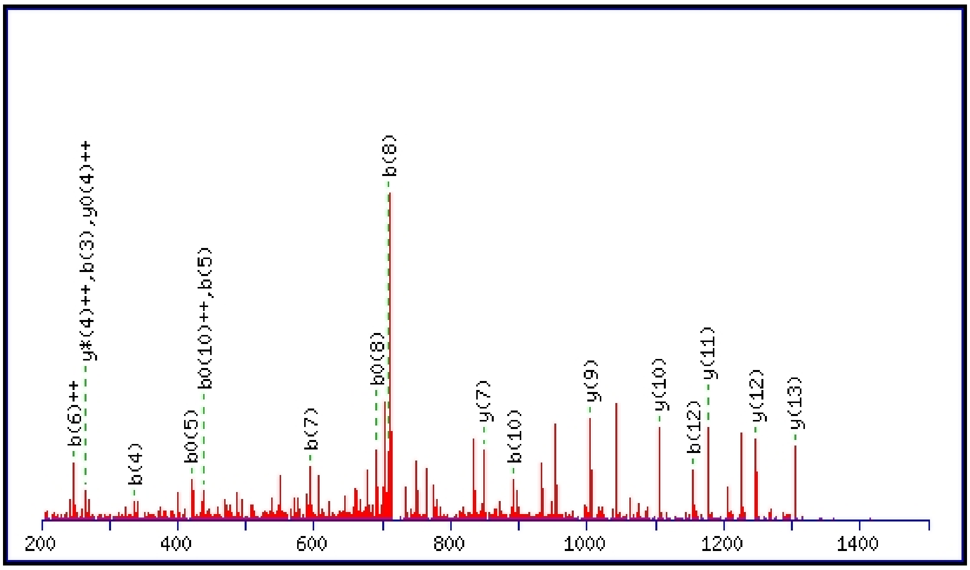
b(6)++
y*(4)++,b(3),y0(4)++
b(4)
b0(5)
b0(10)++,b(5)
b(7)
b0(8)
b(8)
y(7)
b(10)
y(9)
y(10)
b(12)
y(11)
y(12)
y(13)
200
400
600
800
1000
1200
1400

(g)

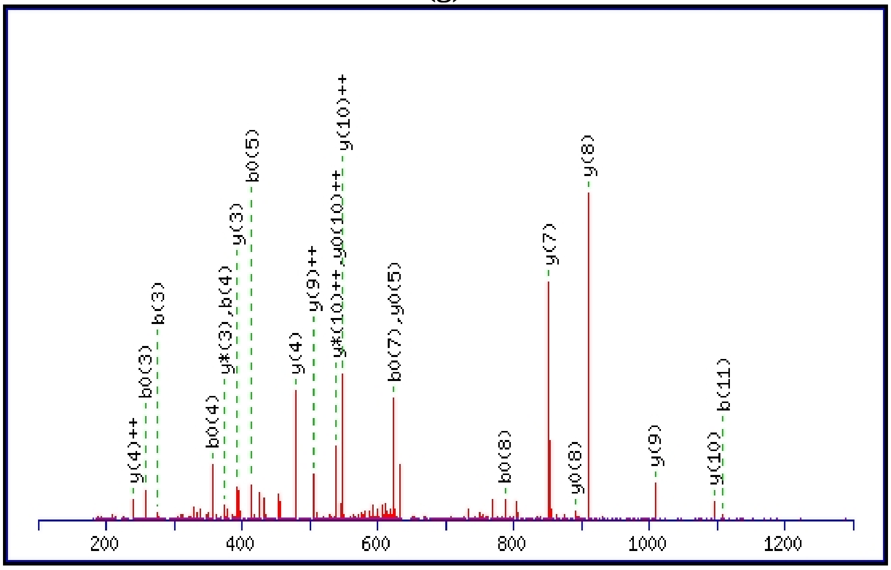
y(4)++
b0(3)
b(3)
b0(4)
y*(3),b(4)
y(3)
b0(5)
y(4)
y(9)++
y*(10)++,y0(10)++
y(10)++
b0(7),y0(5)
b0(8)
y(7)
y0(8)
y(8)
y(9)
y(10)
b(11)
200
400
600
800
1000
1200

(h)

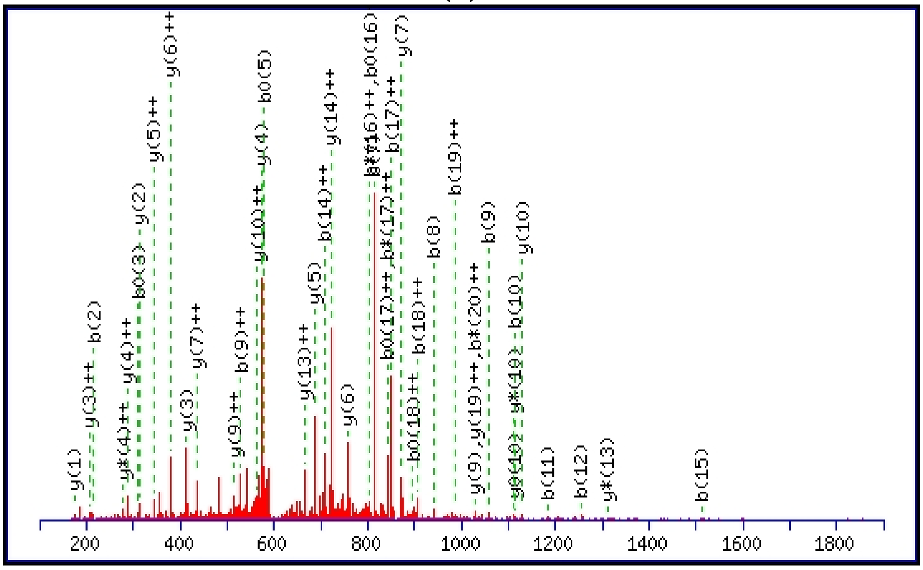
y(1)
y(3)++
b(2)
y*(4)++
y(4)++
b0(3)
y(2)
y(5)++
y(6)++
y(3)
y(7)++
y(9)++
b(9)++
y(10)++
y(4)
b0(5)
y(13)++
y(5)
b(14)++
y(14)++
y(6)
b0(17)++
b(17)++
y(7)
b(18)++
b0(18)++
b(8)
b(19)++
y(9),y(19)++,b*(20)++
b(9)
b(10)
y(10)
b(11)
b(12)
y*(13)
b(15)
200
400
600
800
1000
1200
1400
1600
1800

(i)

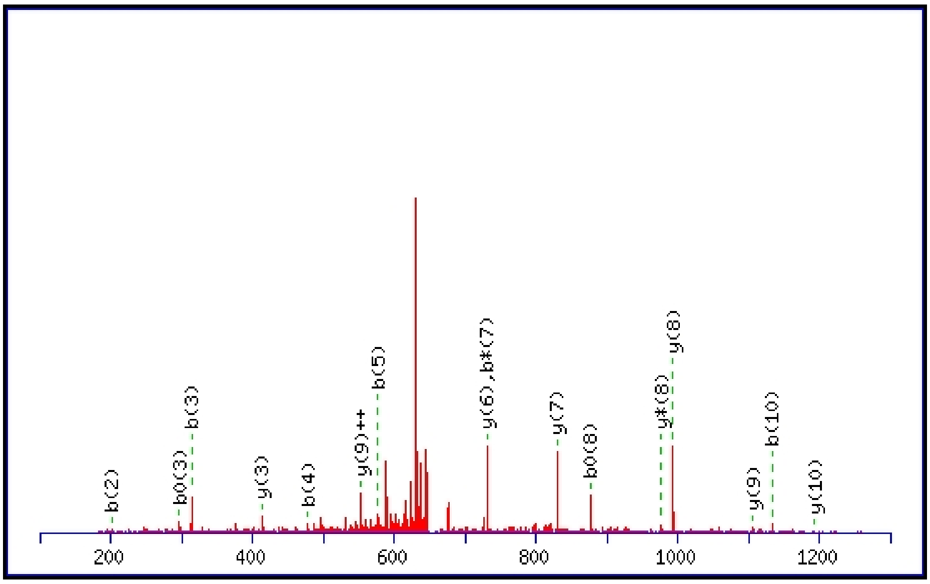
b(2)
b0(3)
b(3)
y(3)
b(4)
y(9)++
b(5)
y(6),b*(7)
y(7)
b0(8)
y*(8)
y(8)
y(9)
b(10)
y(10)
200
400
600
800
1000
1200

(j)

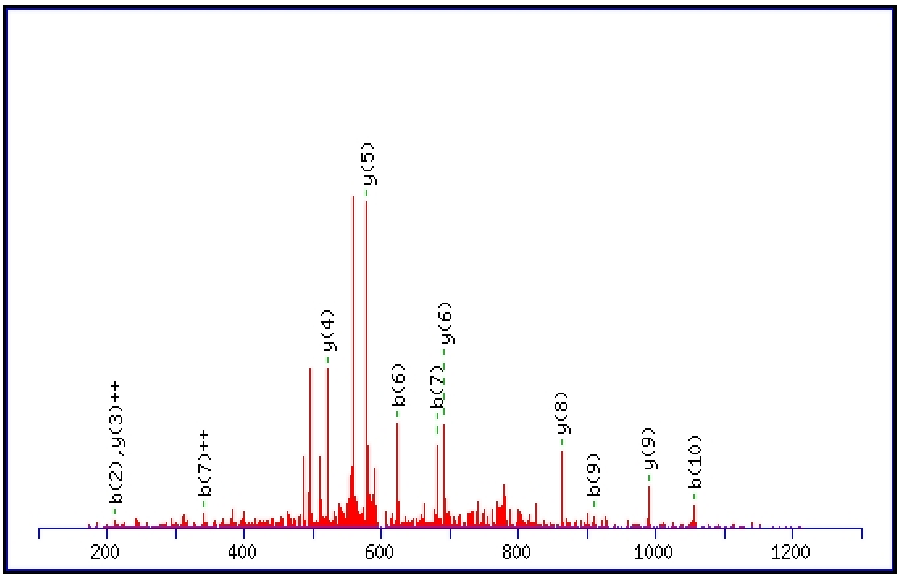
b(2),y(3)++
b(7)++
y(4)
y(5)
b(6)
b(7)
y(6)
y(8)
b(9)
y(9)
b(10)
200
400
600
800
1000
1200

(k)

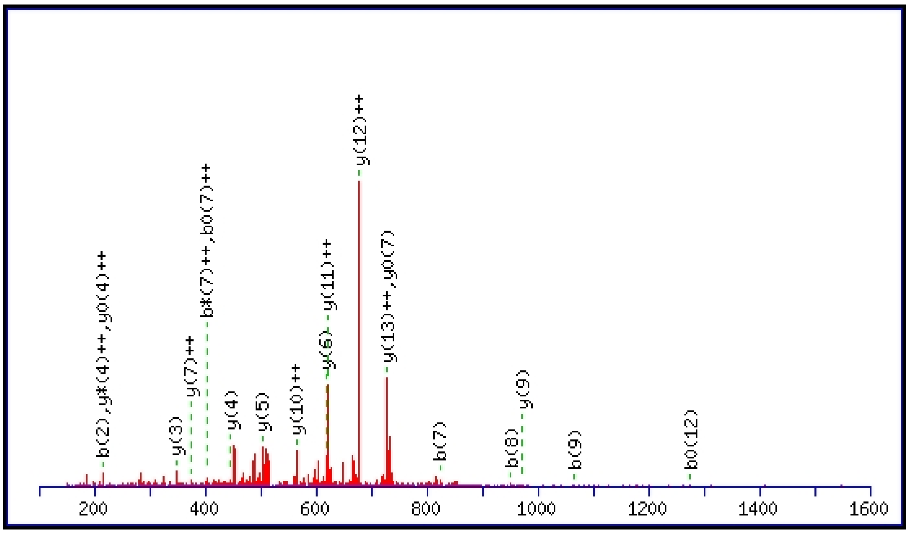
b(2),y*(4)++,y0(4)++
y(3)
y(7)++
b*(7)++,b0(7)++
y(4)
y(5)
y(10)++
y(6)
y(11)++
y(12)++
y(13)++,y0(7)
b(7)
b(8)
y(9)
b(9)
b0(12)
200
400
600
800
1000
1200
1400
1600

(l)

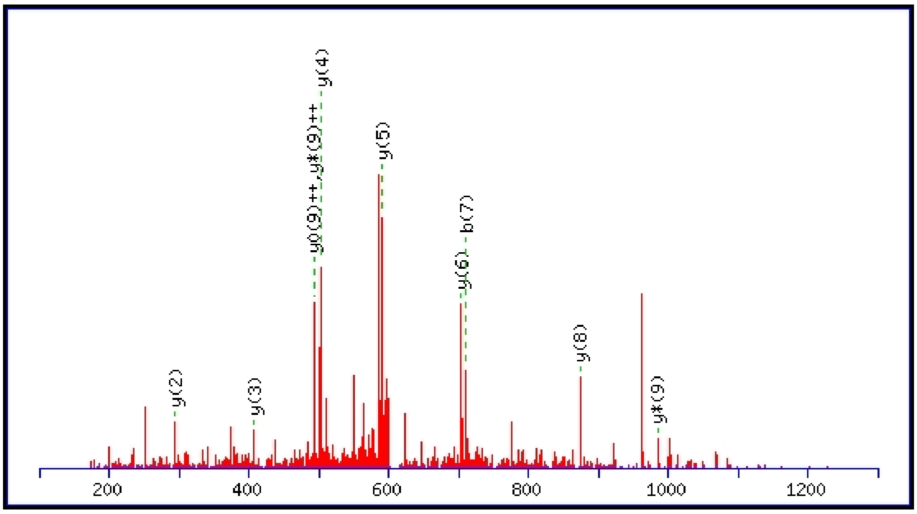
y(2)
y(3)
y0(9)++,y*(9)++
y(4)
y(5)
y(6)
b(7)
y(8)
y*(9)
200
400
600
800
1000
1200

(m)

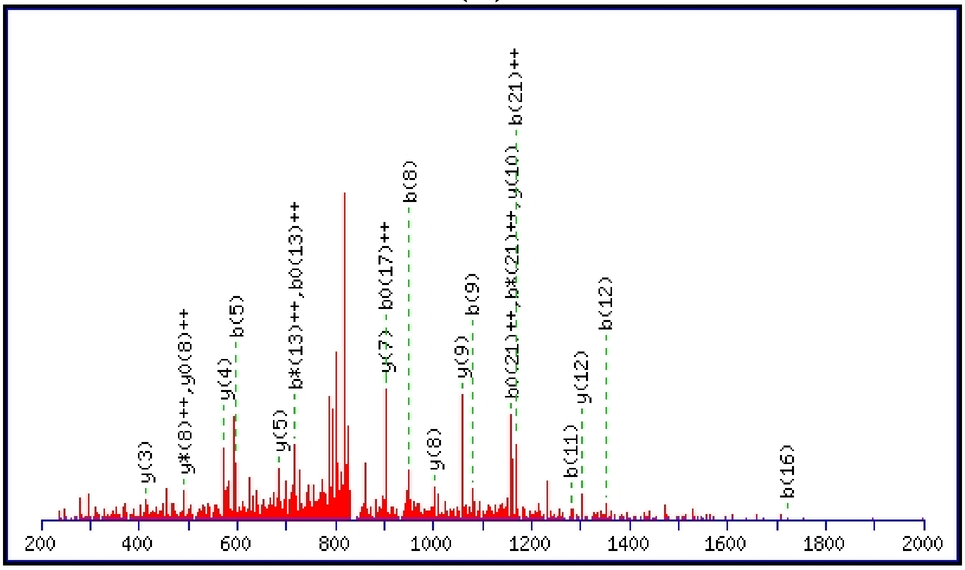
y(3)
y*(8)++,y0(8)++
y(4)
b(5)
y(5)
b*(13)++,b0(13)++
y(7)
b0(17)++
b(8)
y(8)
y(9)
b(9)
b0(21)++,b*(21)++,y(10)
b(21)++
b(11)
y(12)
b(12)
b(16)
200
400
600
800
1000
1200
1400
1600
1800
2000

(n)

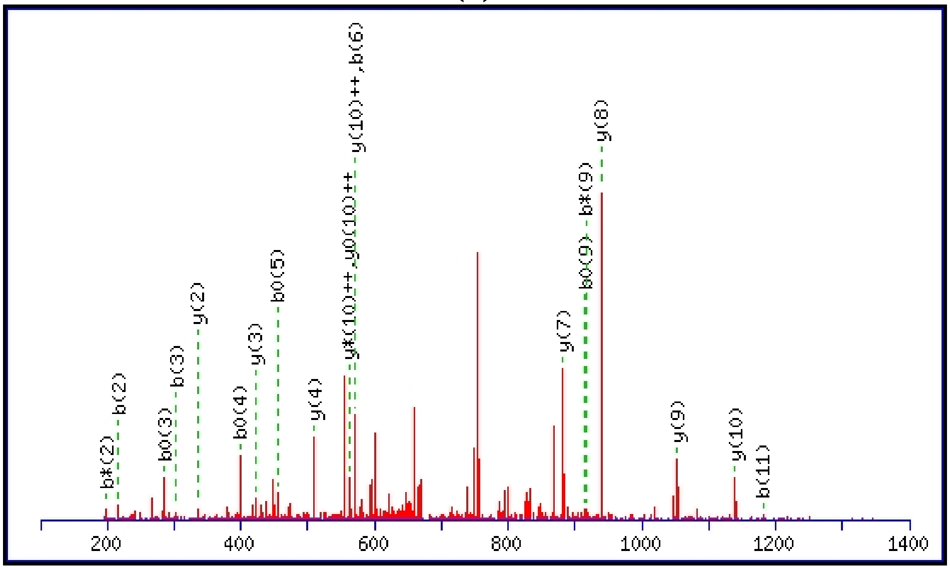
b*(2)
b(2)
b0(3)
b(3)
y(2)
b0(4)
y(3)
b0(5)
y(4)
y*(10)++,y0(10)++
y(10)++,b(6)
y(7)
b0(9)
b*(9)
y(8)
y(9)
y(10)
b(11)
200
400
600
800
1000
1200
1400

(o)

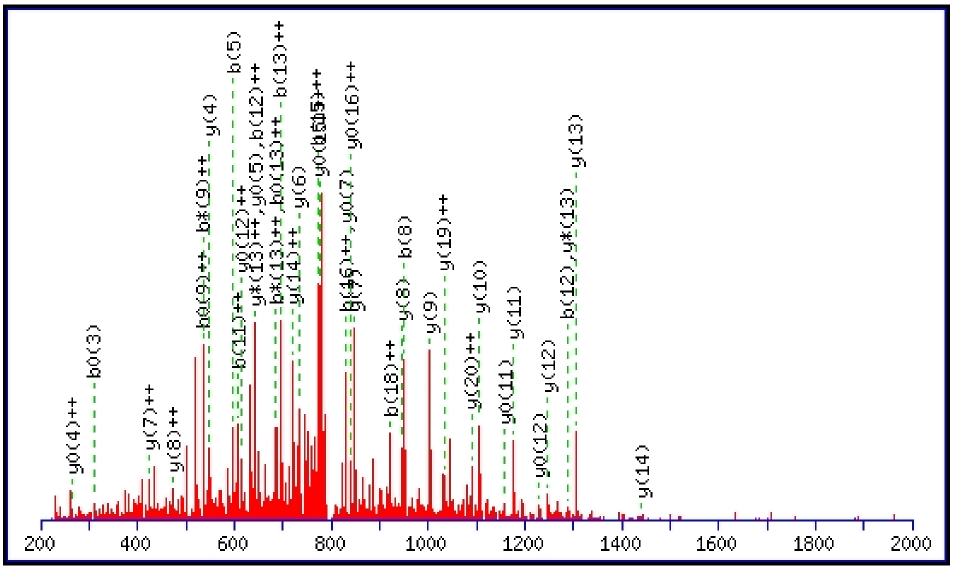
y0(4)++
b0(3)
y(7)++
y(8)++
y(4)
b(5)
y(6)
y0(16)++
b(18)++
y(8)
b(8)
y(9)
y(19)++
y(20)++
y(10)
y0(11)
y(11)
y0(12)
y(12)
b(12),y*(13)
y(13)
y(14)
200
400
600
800
1000
1200
1400
1600
1800
2000

(p)

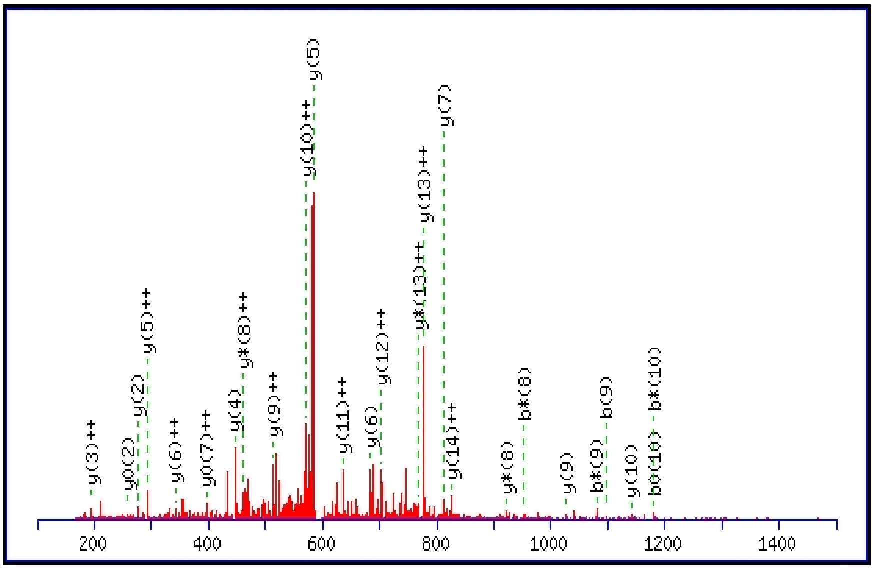

© 2012 by the authors; licensee MDPI, Basel, Switzerland. This article is an open access article distributed under the terms and conditions of the Creative Commons Attribution license (http://creativecommons.org/licenses/by/3.0/).
